# Reduction of radiofrequency induced implant heating via flexible metasurface shielding at 7 T

**DOI:** 10.1016/j.jmr.2025.107918

**Published:** 2025-06-02

**Authors:** Paul S. Jacobs, Wyger M. Brink, Pradnya Narvekar, Neil E. Wilson, Anshuman Swain, Neeraj Panchal, Samir Mehta, Mark A. Elliott, Ravinder Reddy

**Affiliations:** a Center for Advanced Metabolic Imaging in Precision Medicine, Department of Radiology, University of Pennsylvania, Philadelphia, PA, USA; b Magnetic Detection and Imaging group, Techmed Centre, University of Twente, Enschede, the Netherlands; c Department of Oral and Maxillofacial Surgery, University of Pennsylvania, PA, USA; d Department of Orthopedic Surgery, University of Pennsylvania, Philadelphia, PA, USA

**Keywords:** Ultra-high field, Implant, Safety, SAR, Metasurface

## Abstract

Passive implanted devices are commonly contraindicated at ultra-high field MRI due to the risk of radiofrequency heating. Mitigation of this risk has come in many forms, such as modifying implant materials or creating novel radiofrequency coils. These methods require substantial involvement from manufacturers and may not benefit patients with existing implants. In this study, a tailored metasurface design is demonstrated to improve implant safety at 7 T by shielding the local ${\text{B}}_{1}^{+}$ field. A prototype metasurface was designed and implemented with a unit cell size of 15 mm using discrete capacitors of 30 pF values. Phantom and human body model simulations were used to validate differences in the SAR distribution with and without the metasurface. Fiber optic temperature probes were used to measure temperature increase across two representative orthopedic screws placed inside a tissue mimicking phantom during a high-SAR sequence. Phantom and in-vivo imaging were performed to assess the metasurface effect on image quality. With the metasurface, an average maximum temperature decrease of 0.50 °C or 34.9 % near the implant was observed. RF field simulations yielded similar decreases in SAR for the phantom (40.7 %) and substantial decreases for the in-vivo leg model (97 %). Phantom image SNR showed a global 8.5 % decrease with the metasurface while in-vivo images showed a 4.8 % decrease in SNR, with the region in its immediate vicinity experiencing substantial signal drop. These results demonstrate the feasibility of a metasurface designed to substantially reduce local RF induced heating with only minor degradation of image quality. Future work will focus on refinement of the metasurface design and further in-vivo testing.

## 1. Introduction

MRI implant compatibility has been an increasing research focus since the introduction of higher field strength systems. With ultra-high field MRI (≥7 T) now becoming more available, ensuring the safety of patients with medical implants during scans has become even more important. This type of diagnostic imaging can be beneficial for many patients who have undergone an invasive surgical procedure, for example in the case of post operative monitoring of neurosurgical tumor resection [1] where orthopedic implants have been placed [2]. Many of these procedures result in the placement of metallic implants such as cranial fixation plates or, in the case of orthopedic surgeries, bone screws and plates. Furthermore, patients who have these metallic implants are typically in need of more consistent follow-up imaging in the tissue region near the implant site. However, due to the safety risks associated with imaging patients where the implant may be in a strong radiofrequency (RF) field, most patients are unable to benefit from MR imaging.

The main safety risk comes from the potential heating of tissue around the implant that occurs when the metallic material interacts with the RF field generated by the RF transmit coil [3]. In particular, the RF magnetic field (B_1_) produced by the transmit coil can induce current inside conductive implants and lead to a perturbed scattered *E*-field around the implant which can modify the RF power deposition in the surrounding tissue [4]. In the current work, the second mechanism is of primary interest as it is the main source of heating. The phenomenon originates from the electrically conductive nature of a metallic implant in which the RF transmit field induces currents on the implant [5]. These currents travel along the implant and perturb the native *E*-field to create a scattering effect, typically with maxima around the distal ends of the implants, resulting in electrical currents in the surrounding tissue with associated resistive heating and increases in the specific absorption rate (SAR). The relationship between the SAR and *E*-field is described in Eq. 1 below, where regulatory limits on local RF power absorption are defined in terms of 10 g or 1 g-averaged SAR by the FDA. Here, the additional terms represented in the equation are electrical conductivity (σ) and density (ρ).


(1) $$SAR\equiv \frac{\sigma |\vec{E}{|}^{2}}{2\rho }$$

In the worst-case scenario, when the length of these types of implants approaches λ/2 in length (where λ represents the wavelength of the RF transmit field in the surrounding tissue), they can exhibit a resonance effect (also referred to as the “antenna effect”) which greatly amplifies the scattered *E*-field at their distal ends. This amplifies the current in the surrounding tissue which further increases local RF-induced heating [6]. It should be noted that this effect is dependent on the orientation of the implant relative to the E-field. A parallel orientation will produce substantial more heating compared to a perpendicular orientation [3].

In the past there have been reported adverse events associated with implant related heating during MR imaging [7]. Current mitigation strategies for minimizing the amplified heating effects caused by these implants include modifying the background *E*-field the implant is exposed to or the scattered *E*-field generated from the implant itself. Several studies have been performed with the aim of modifying the background *E*-field by using reconfigurable RF coils [8–11]. Work has also been done to modify the design of DBS and pacemaker leads [12–15], stents [16], orthopedic screws [17], and hip implants [18] in an effort to make them more compatible with MRI systems. However, these solutions call for substantial hardware modifications from the coil and implant manufacturers. Parallel transmit (pTx) methods have also been developed with the goal of tailoring the transmit pulses to minimize the local SAR around a DBS lead at 3 T [19]. However, sequence translation from single channel transmit to pTx can be complex, associated hardware can be expensive and not widely available, and pTx SAR limitations are often highly restrictive. Additionally, since most medical device manufacturers do not yet perform safety testing at ultra-high field strengths, most patients with electrically conductive implants are simply denied access to imaging at these field strengths.

In recent years, there has been an increase in the development and utilization of metasurfaces to tailor the RF transmit field. These passive devices are 2-dimensional sheets containing periodic unit cell structures made from composite materials, typically tailored towards enhancement of the MR signal [20–22]. Based on the Kerker effect, they function by altering the electric and magnetic responses leading to a redistribution of the RF electric and magnetic fields within the sample. Typically, this property has been exploited to increase forward scattering, corresponding to the first Kerker condition. This can be used, when coupled with an electric dipole emitter, towards spatial enhancement of the signal near these surfaces leading to improved image quality [23,24]. Metasurfaces can also be adjusted to minimize forward scattering and increase backscattering, corresponding to the second Kerker condition. This is a well-known general characteristic of metamaterials but has rarely ever been given a practical utilization [25]. Dubois et al. [23] was able to demonstrate both Kerker conditions by showing ${\text{B}}_{1}^{+}$ enhancement as well as reduction depending on the length of wire used in a “hybrid meta-atom”, corresponding to shifts in the effective phase of the fields produced. This region of reduced RF field magnitude directly adjacent to the metasurface results in an effectively eliminated B_1_ and *E*-field. While there have been some early implementations of this concept in the form of secondary resonators [26] or full conductive shields [27] many of them have not been assessed at field strengths higher than 3 T (corresponding to Larmor frequencies below 128 MHz) or with in vivo image quality effects in mind.

Therefore, while most studies focus on designing metasurfaces that provide image enhancement, the goal of this work is to evaluate the feasibility of using a metasurface tailored to attenuate the *E*-field and reduce RF heating observed around orthopedic metallic implants in a 7 T MRI while also providing an initial evaluation of phantom and in-vivo calf image quality across the effective range of the device.

## 2. Theory

**Fig. 1.**
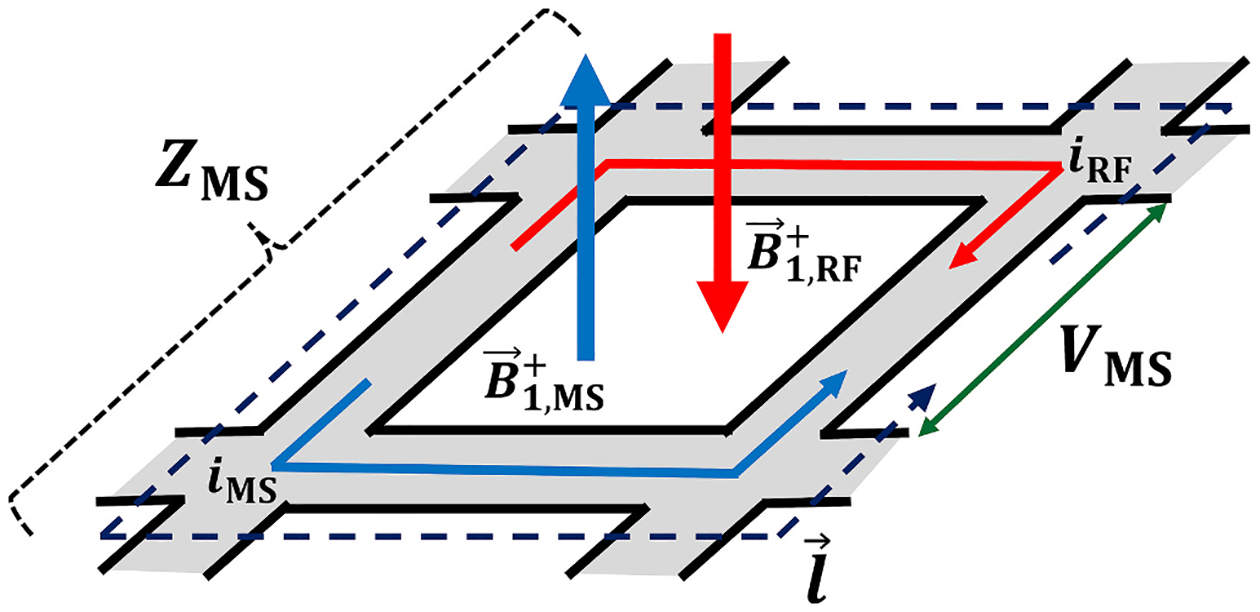
Unit cell diagram: A schematic showing an example metasurface unit cell, and the mechanism of primary RF transmit field (${\text{B}}_{1,\text{RF}}^{+}$) induced currents (i_RF_) and secondary metasurface induced (i_MS_) currents along the gray conductor trace path (l). These currents induce a voltage potential (V_MS_) across the unit cell, resulting in a secondary field (${\text{B}}_{1,\text{MS}}^{+}$). The phase difference between the primary and secondary field is determined by the characteristic impedance (Z_MS_) of the unit cell.

One method for describing a metasurface is in the form of an equivalent circuit model, seen in Fig. 1, which is a fast and intuitive method for presenting the physical characteristics of the device [26,28]. In this description, the magnetic field produced from the RF transmit coil ($B_{1,\text{RF}}^{+}$) couples into individual unit cells of the metasurface and induces a voltage potential ($V_{\text{MS}}(t)$) across each of the unit cell’s capacitors, which is integrated along the conductor path (l), as seen in Eq. 2 below.


(2) $$V_{\text{MS}}(t)=-\frac{d}{d\text{t}}\oint_{\vec{l}}{\vec{B}}_{1,\text{RF}}^{+}(t)•d\vec{l}+f(\vec{E})$$

It should be noted that *E*-field will also have an influence on the voltage potential, which is reflected by the general function term, $f(\vec{E})$. A current density is then induced on the metasurface, proportional to V_MS_(t), which in turn produces a secondary magnetic field ($B_{1,\text{MS}}^{+}$), described as

(3) $${\vec{B}}_{1,\text{MS}}^{+}(t)\propto i_{\text{MS}}(t)=\frac{V_{\text{MS}}(t)}{Z_{\text{MS}}}$$

which also results in a corresponding change in the local *E*-field, i.e.


(4) $$\nabla \times \vec{E}=-\frac{\partial {\vec{B}}_{1}}{\partial t}$$

The characteristic impedance of the metasurface ($Z_{\text{MS}}$) can be written as

(5) $$Z_{MS}=R_{S}+jX$$

As noted, it is assumed in the calculations that the real component of the metasurface impedance (R_S_) is a small non-zero quantity. The characteristic impedance is the result of a passive electromagnetic response of the metasurface design, which determines not only the magnitude of the response but influences also the phase (φ) of $B_{1,\text{MS}}^{+}$, described in Eq. 6.


(6) $$\varphi ={\text{tan}}^{-1}\left(\frac{Im\left(Z_{\text{MS}}\right)}{Re\left(Z_{\text{MS}}\right)}\right)$$

We note that when the characteristic impedance of the metasurface (Eq. 5) falls into the inductive regime, where the reactance (X) is positive, the secondary $B_{1,\text{MS}}^{+}$ field will be out of phase with the primary $B_{1,\text{RF}}^{+}$, resulting in a reduction in the total $B_{1}^{+}$ field magnitude in the immediate vicinity of the device.

This principle can also be extended towards a metasurface that produces a secondary magnetic field with the same phase as $B_{1,RF}^{+}$, resulting in a $B_{1}^{+}$ enhancement of the RF field in the immediate vicinity, as demonstrated in our previous work [21]. This corresponds to a metasurface with a characteristic impedance that falls within the capacitive regime and is also reflected later in the results.

## 3. Methods

### 3.1. Metasurface design and optimization

**Fig. 2.**
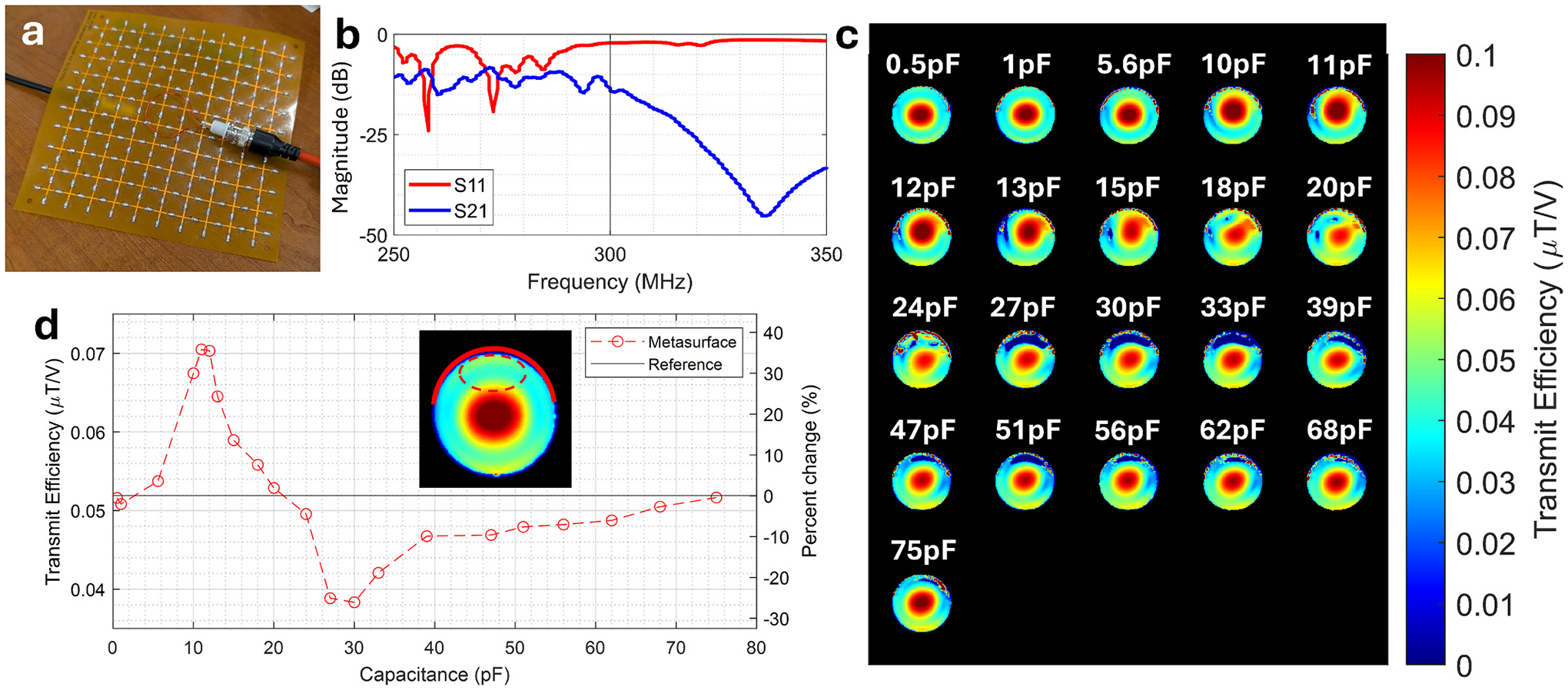
Metasurface characterization experiment: An illustration showing (a) the physical prototype of the single layer PCB used in optimization experiments with one untuned loop probe shown as used to measure the S_11_ parameter approximately in the PCB center. (b) Reflection (S_11_) and transmission (S_21_) coefficient measurements acquired for the optimized metasurface configured with 30 pF capacitors. (c) Phantom transmit efficiency maps (${\text{B}}_{1}^{+}$ maps normalized to the corresponding acquisition transmit reference voltage) acquired at the axial center of the phantom for each capacitor configuration can be seen, showing the direct effect of the metasurface on the field distribution which is also quantified graphically at the bottom left (d) within the ROI shown (red dashed line) in the figure inset. We note that the solid red curve present in the same inset reflects the physical placement location of the metasurface.

The metasurface design chosen here is based on a frequency selective surface, specifically a mesh grid [22]. The design was also used in a previous study by our group [21] towards the development of a metasurface configuration that would enhance the ${\text{B}}_{1}^{+}$ field and improve image quality. Empirical optimization was performed via physical testing of a prototype 18 cm × 18 cm flexible printed circuit board (PCB) copper mesh grid metasurface of thickness 83.5μm (polyimide dielectric substrate), copper trace width of 1 mm and a 15 mm × 15 mm standard unit cell size, as seen in Fig. 2a. The PCB was implemented with 220 evenly distributed discrete capacitors in 21 steps (0.5, 1, 5.6, 10, 11, 12, 13, 15, 18, 20, 24, 27, 30, 33, 39, 47, 51, 56, 62, 68, 75 pF). Each of these capacitor configurations were tested with the metasurface attached to the top of a cylindrical saline phantom (20 cm height, 6 cm radius, 5 g/L NaCl, 3.75 g/L NiSO_4_, $\sigma =2\;\text{mS}/\text{m},\varepsilon_{\text{r}}=74$) to observe changes in the ${\text{B}}_{1}^{+}$ field. A single ROI immediately adjacent to the center of the metasurface was chosen to quantify the average transmit efficiency to assess the direct local effect of the metasurface in the expected region. The power reflection and transmission coefficients of the metasurface were quantified by measuring the scattering parameters (S-parameters), S_11_ and S_21_ respectively, using a pair of untuned loop probes (33 mm diameter) placed 1 cm away from the center and on opposite sides of the unloaded metasurface using an Agilent E5061A Network Analyzer (Keysight Technologies, Santa Clara, CA, USA).

### 3.2. Measurement configuration and phantom composition

**Fig. 3.**
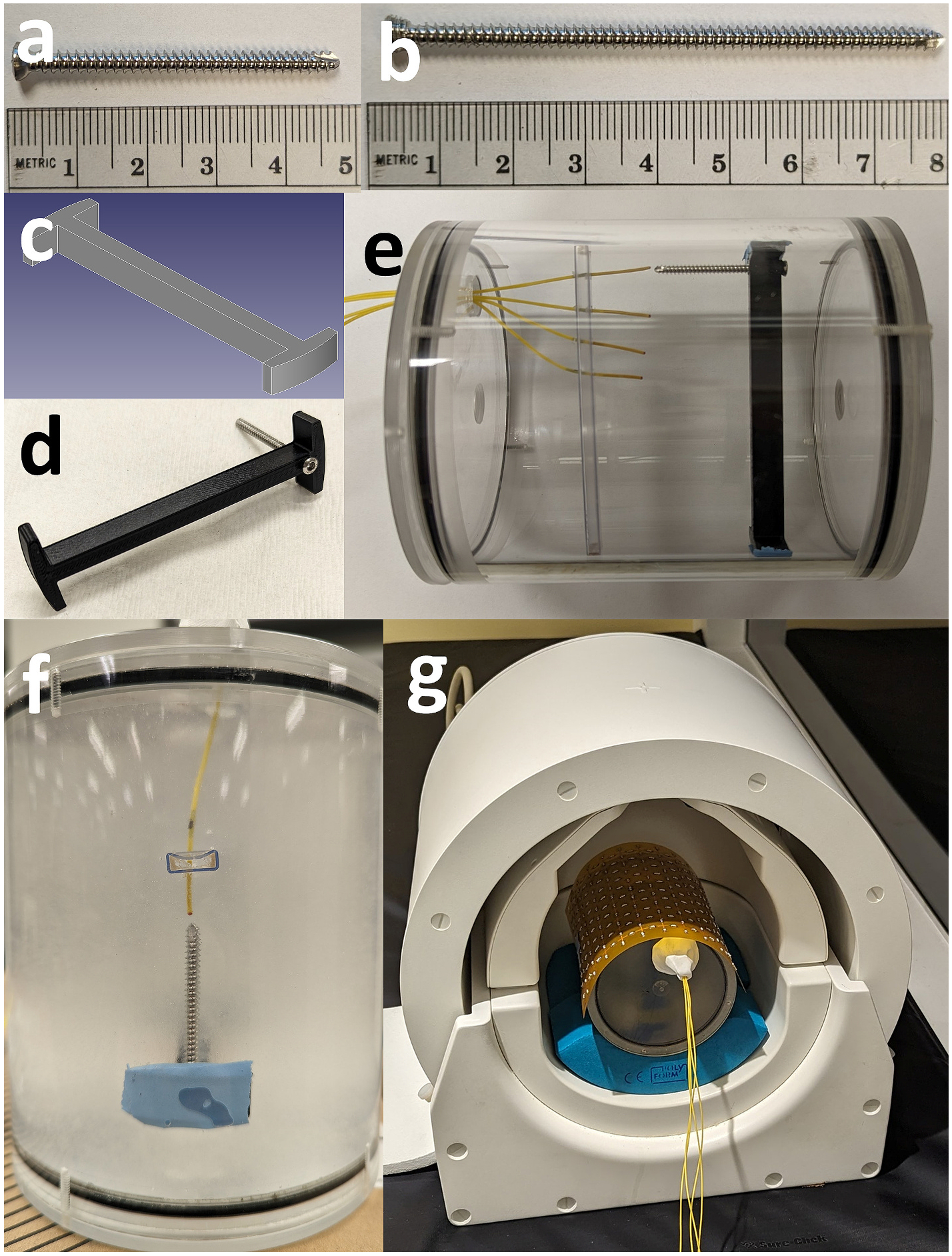
(a) The 46 mm and (b) 76 mm orthopedic screws used in the empirical phantom measurements. (c) A CAD model of the plastic mount designed to hold the implants inside the phantom. (d) The physical plastic mount with an orthopedic screw placed in it. (e) A photo showing the placement of the fiber optic temperature probes inside the empty phantom and their relative separation distance from each other (10 mm). (f) The first fiber optic probe can be seen placed in a PAA phantom with the tip approximately 2 mm away from the screw tip. (g) The phantom assembly placed inside the Nova head coil for testing.

The two orthopedic screw implants used for the phantom temperature measurements were a 46 mm and 76 mm stainless steel self-tapping cortex screws (Depuy Synthes, Warsaw, IN, USA) (Fig. 3a and b). A custom 3D-printed mount was designed (Fig. 3c and d) to hold the implants at the center of a phantom approximately 20 mm away from the metasurface; which was attached to the top of the phantom above each of the implants. Each implant-holder assembly was placed inside a prepared cylindrical (100 mm diameter, 120 mm height) tissue mimicking polyacrylic acid (PAA) phantom (10 g/L PAA, 1.32 g/L NaCl, 800 mL H_2_O) [29]. Four fiber optic temperature probes (FOB104, Omega Engineering, Norwalk, CT, USA) were placed inside the phantom, with the first placed close to the distal end of each implant at a distance from the tip of approximately 2 mm, as seen in Fig. 3f. The three additional fiber optic probes were placed at 10 mm intervals inside the phantom relative to the first, as seen in Fig. 3e. The phantom was tested with each implant, with and without the metasurface present and additionally, without an implant present as a reference comparison. The heating experiments were repeated in three trials per implant and metasurface condition with average temperature values being recorded once every second. Experimental replicates were collected on different days with full repositioning of the RF coil and metasurface, to evaluate reproducibility. The implant and temperature probes remained in place inside the phantom for the duration of the replicate experiments.

### 3.3. Phantom MR acquisition and image processing

All experiments were performed using a 7 T MRI (MAGNETOM Terra, Siemens Healthcare, Erlangen, Germany), in which the orthopedic implants were tested using a single-channel transmit/28-channel receive phased array knee coil (Quality Electrodynamics, Mayfield Village, OH, USA). A ${\text{B}}_{1}^{+}$ mapping sequence, described by Volz et al. [30], was used for metasurface characterization with the following parameters: shot TR/TE = 7400/1.46 ms, 5 shots, preparation flip-angles = 20°/40°/80°, field-of-view = 240 mm × 204 mm, in-plane resolution = 1 mm × 1 mm, slice thickness = 2 mm, number of slices = 12. Transmit efficiency maps were generated from resulting B_1_ maps by normalizing to the transmit reference voltage during each experimental acquisition. To achieve a long duration (20-min) high-SAR (>95 %) heating protocol, a gradient echo (GRE) sequence was used with the nominal parameters: TR/TE = 11/6 ms. To maximize SAR, the flip angle was set to a constant 90° and the RF coil reference voltage was increased until SAR was above 95 %. These parameters were then used between both metasurface and reference experimental conditions which showed the same maximum SAR level. To maximize energy input, all sequences were run in first-level operating mode.

To assess basic phantom image quality for metasurface and implant combinations, single slice dual-echo GRE images were acquired with the following parameters: TR = 200 ms, TE_1_ = 2.99 ms, TE_2_ = 5.79 ms, flip-angle = 30°, matrix size = 240 × 210, in-plane resolution = 1 mm × 1 mm, slice thickness = 2 mm. The implant used in these imaging studies was the 46 mm self-tapping orthopedic screw seen in Fig. 3f. Image signal-to-noise ratio (SNR) was calculated as the ratio of the average signal over the entire phantom cross-section (ROI 1) and the standard deviation of the background noise (calculated as the average noise in the four corner regions of the image, seen in Fig. 7b). SNR was also quantified in an additional smaller ROI area (referred to as ROI 2) positioned approximately 20 mm posterior to the metasurface placement, outside its effective range, to quantify the change in SNR in the remainder of the phantom. All phantom images were processed in MATLAB (The Math-works, Natick, MA, USA).

### 3.4. Electromagnetic simulations

A full wave finite-difference time domain (FDTD) solver (Sim4Life version 8.0, ZMT, Switzerland) was used to numerically simulate the SAR distribution with and without the metasurface wrapped around a simplified cylindrical implant model inside of the cylindrical PAA phantom, as seen in Fig. 5a. A resonant model of the RF transmit coil was tuned to 300 MHz using lumped elements in the simulation grid. The PAA phantom, including its outer acrylic phantom casing, were modelled using the corresponding dielectric properties (PAA; $\sigma =0.517\;\text{S}/\text{m},\varepsilon_{\text{r}}=82.12$) [29]. A generic rod (42.5 mm length, 4 mm diameter) was assigned to the electrical properties of stainless-steel and placed inside the phantom 20 mm away from the phantom surface, as seen in Fig. 5b. The combined phantom and implant model were placed in the center of the coil model. The curved metasurface was modelled by importing the PCB design and assigning 220 discrete 30 pF capacitors at the associated locations. The metasurface conductor traces were assigned as perfect electric conductors and the PCB substrate material as polyimide $\left(\sigma =60\;\mu \text{S}/\text{m},\varepsilon_{\text{r}}=3.50\right)$. The metasurface model was then positioned 0.5 mm above the phantom model and within the center of the RF coil. Phantom simulations were performed with and without the metasurface using a nominal spatial resolution of 0.8 mm over the phantom, stainless-steel rod, and RF coil model, and took approximately 3 h to execute. As a comparison, a simulation using a copper sheet (continuous conductor) of identical size and placement without an implant present was performed using the same PAA phantom and RF coil, as seen in Fig. 6a. Additionally, a 1-dimensional line profile was used which ranged from the top to bottom of the phantom to plot the ${\text{B}}_{1}^{+}$ and *E*-field values as a function of phantom depth for both this and the original metasurface simulation without an implant. An example showing the orientation of the line profile can be seen in Fig. 6b. Additional simulations using a human leg model (Duke v3.0, IT’IS Foundation) were also performed with the same stainless-steel rod placed at the geometric center of the metasurface and RF coil model, partially embedded in the anterior tibia bone to mimic a realistic implant placement. The leg model, metasurface placement, and implant placement can be seen in Fig. 7a, b, and c. SAR_1g_ maps were generated with and without the metasurface present. These simulations were performed using a nominal spatial resolution of 1 mm over the leg and stainless-steel rod, with both simulations taking approximately 6 h to execute.

All simulations were set to reach a convergence of −50 dB and were performed on a dedicated GPU (GeForce RTX 4080, NVIDIA, USA). In both phantom and human leg models, the data post-processing consisted of adjusting the RF coil to quadrature drive with a normalized excitation power of 1 W. The primary quantity extracted from all simulated results was the spatial 1 g-average SAR (SAR_1g_) as well as the peak spatial SAR (psSAR – maximum value after averaging over any 1 g spatial volume) value.

### 3.5. In-Vivo MR acquisition and processing

To assess the metasurface shielding effect on in-vivo image quality, calf images were acquired on a single healthy volunteer with written informed consent under an approved University of Pennsylvania Institutional Review Board protocol. Images were acquired with and without the metasurface present using a single-channel/28-channel phased array proton knee coil; the same coil used in the empirical phantom testing. Axial ${\text{B}}_{1}^{+}$ maps were acquired and processed into transmit efficiency maps as described previously in phantom experiments. Average and standard deviation transmit efficiency values were calculated across all values within the displayed calf slice. Additionally, axial and sagittal anatomical GRE images were also acquired with the same parameters as previously described in phantom experiments. A 5 mm foam spacer was placed between the subject and metasurface during the acquisition. All in-vivo imaging was performed in normal operating mode. Image SNR was calculated in two local ROIs to assess the metasurface effect outside of its immediate vicinity, as was done in the phantom imaging experiments. The ROI 1 comprised the tissue signal in the entire slice while ROI 2 only included a subregion approximately 2 cm from the top of calf, as seen in Fig. 9a. All in-vivo images were processed in MATLAB.

## 4. Results

### 4.1. Metasurface optimization

Transmit efficiency maps showed that as a function of capacitance, the ${\text{B}}_{1}^{+}$ field distribution, seen in Fig. 2c, goes through pronounced changes. Initially, the maps show a local ${\text{B}}_{1}^{+}$ enhancement between the values of 5.6 pF and 18 pF, followed by field reduction occurring between 24 pF and 68 pF in the immediate vicinity of the metasurface. The maximum amount of field reduction was observed to occur between the values of 27 pF and 30 pF. This indicates that the induced currents in the metasurface, and therefore their associated secondary ${\text{B}}_{1}^{+}$ fields, are maximally out of phase with the primary ${\text{B}}_{1}^{+}$ field at those values. This is also reflected in Fig. 2d where the average transmit efficiency is quantified in an ROI (seen in figure inset) immediately adjacent to the metasurface, as capacitance was varied. With the metasurface configured to 30 pF, the chosen optimum, the average transmit efficiency in the ROI was approximately 0.038μT/V in comparison to 0.052μT/V when the metasurface was absent, corresponding to a decrease of approximately 25 %. Scattering parameter measurements in the optimized metasurface, seen in Fig. 2b, confirms the observed behavior and shows a high degree of power reflection (S_11_) and a low amount of power transmission (S_21_) at 300 MHz.

### 4.2. Time series temperature evaluation

**Fig. 4.**
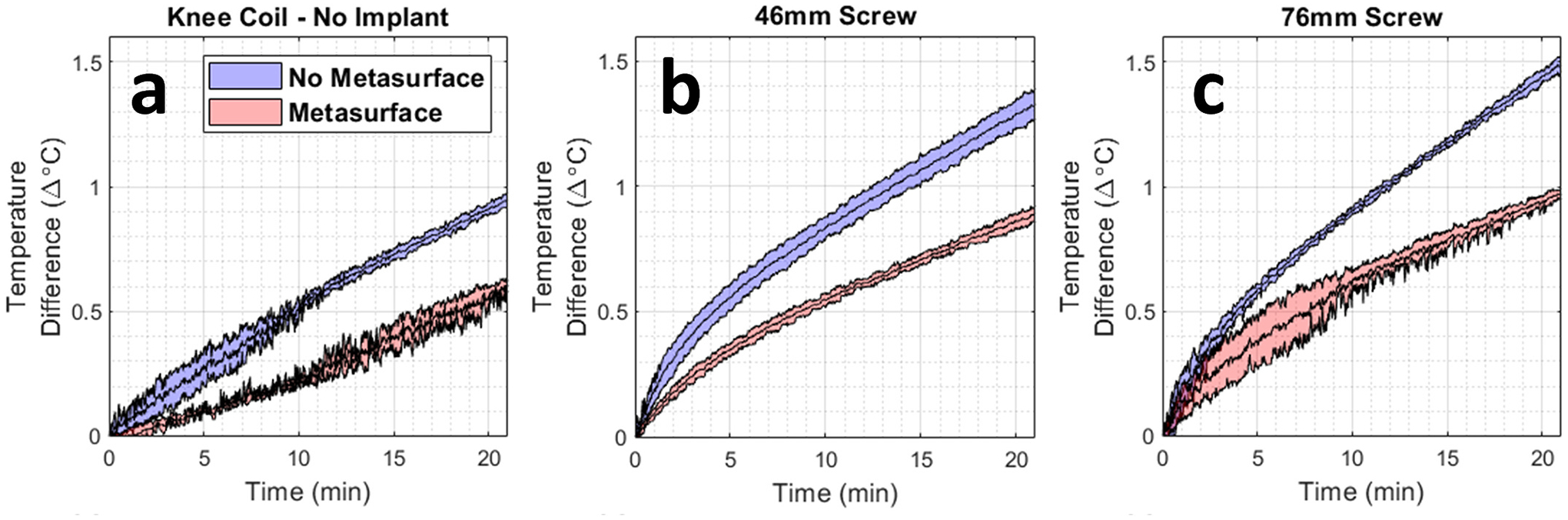
Plots showing the average time-dependent temperature difference across the 20-min heating scan with and without the metasurface present for (a) a reference phantom with no implant present, (b) the 46 mm screw and (c) the 76 mm screw. The shaded regions of the curves represent the standard deviation across three experimental replicates acquired on different dates and times.

Fiber optic temperature measurements showed that the maximum amount of heating occurred in probe 1, which was closest to the implant, while the remaining three probes showed marginal differences. Time series temperature data from probe 1 can be seen in Fig. 4a, b, and c for the reference phantom, 46 mm screw, and 76 mm screw. The rate of temperature increase in the control phantom, 46 mm screw, and 76 mm screw were consistently altered with the metasurface present, reflecting a change in the local *E*-field. This shows that the use of the metasurface resulted in lower RF-induced heating for both implants tested. It also showed that RF-induced heating was reduced in the phantom that did not contain an implant. Quantitatively, the metasurface decreased the observed heating in the control phantom from 0.98 °C to 0.62 °C (36.6 % decrease) while the heating in the 46 mm screw decreased from 1.35 °C to 0.91 °C (32.2 %) and the heating from the 76 mm decreased from 1.53 °C to 0.95 °C (37.6 %).

### 4.3. Simulation results

**Fig. 5.**
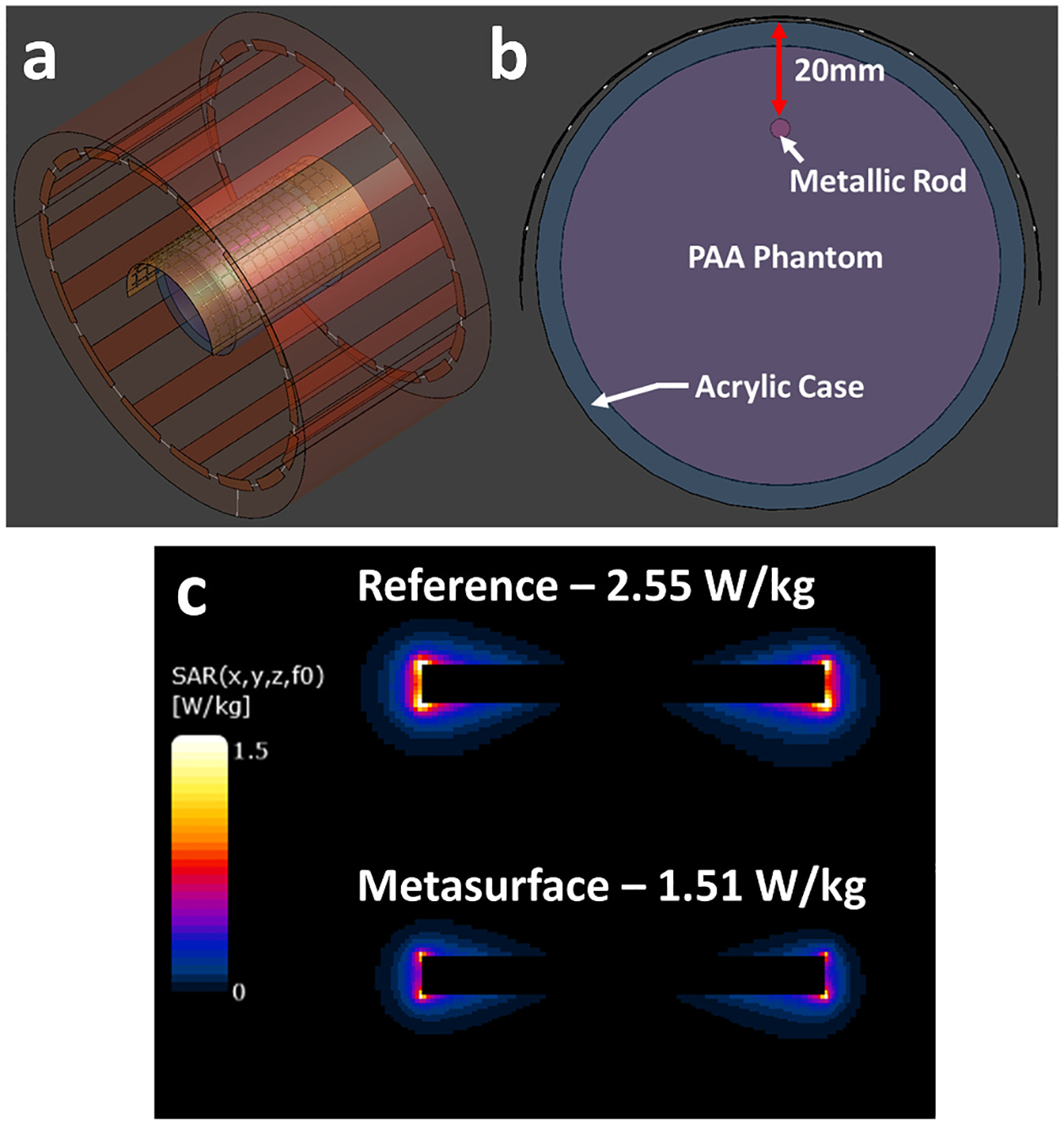
(a) The RF coil, metasurface, phantom, and stainless-steel rod models in the Sim4Life environment with (b) a detailed diagram showing the placement of the stainless-steel rod (pink cylinder) inside the PAA phantom 20 mm below the metasurface. (c) SAR_1g_ maps of the stainless-steel rod can be seen showing a substantial decrease in magnitude near the distal ends. psSAR values are displayed for reference and metasurface simulations. It should be noted that the SAR_1g_ maps are displayed sagittally from the direct center of the simulated phantom and stainless-steel rod.

**Fig. 6.**
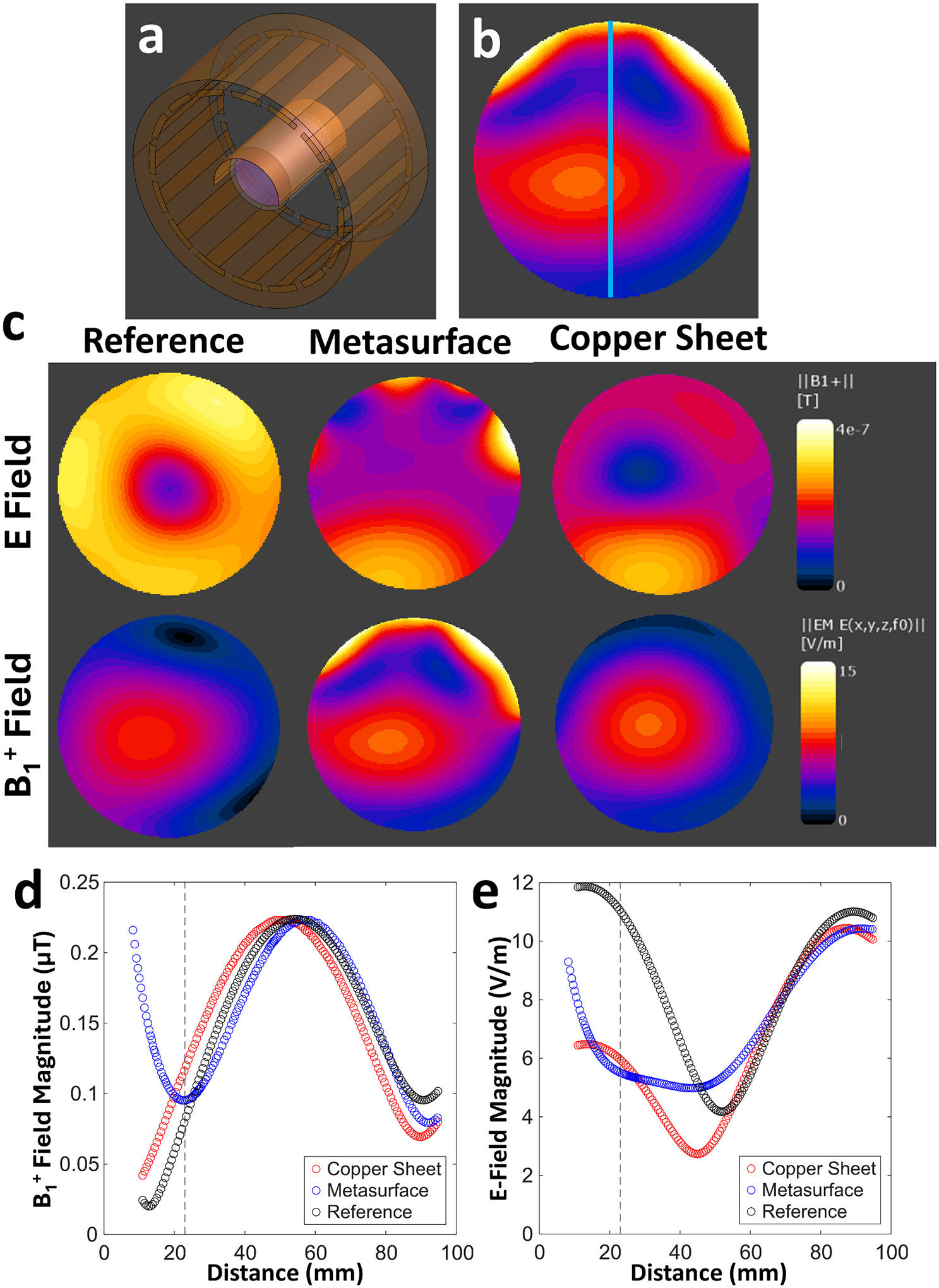
(a) The RF coil, copper sheet (placed in the same position as the metasurface), and phantom models in the Sim4Life environment. (b) A representative image showing a standard line profile (blue line) along which ${\text{B}}_{1}^{+}$ and E field data was plotted as a function of phantom depth for the reference, metasurface, and copper sheet. (c) Simulation results showing ${\text{B}}_{1}^{+}$ and E field maps from a transverse slice displayed at the center of the PAA phantom. (d) A line profile comparison of the simulated ${\text{B}}_{1}^{+}$ field data as a function of phantom depth for all simulated conditions. (e) The corresponding E field data as a function of phantom depth. Dashed lines show that the ${\text{B}}_{1}^{+}$ minimum is located at approximately 23 mm. It should be noted that the distance here was defined as the distance from the metasurface/copper sheet.

**Fig. 7.**
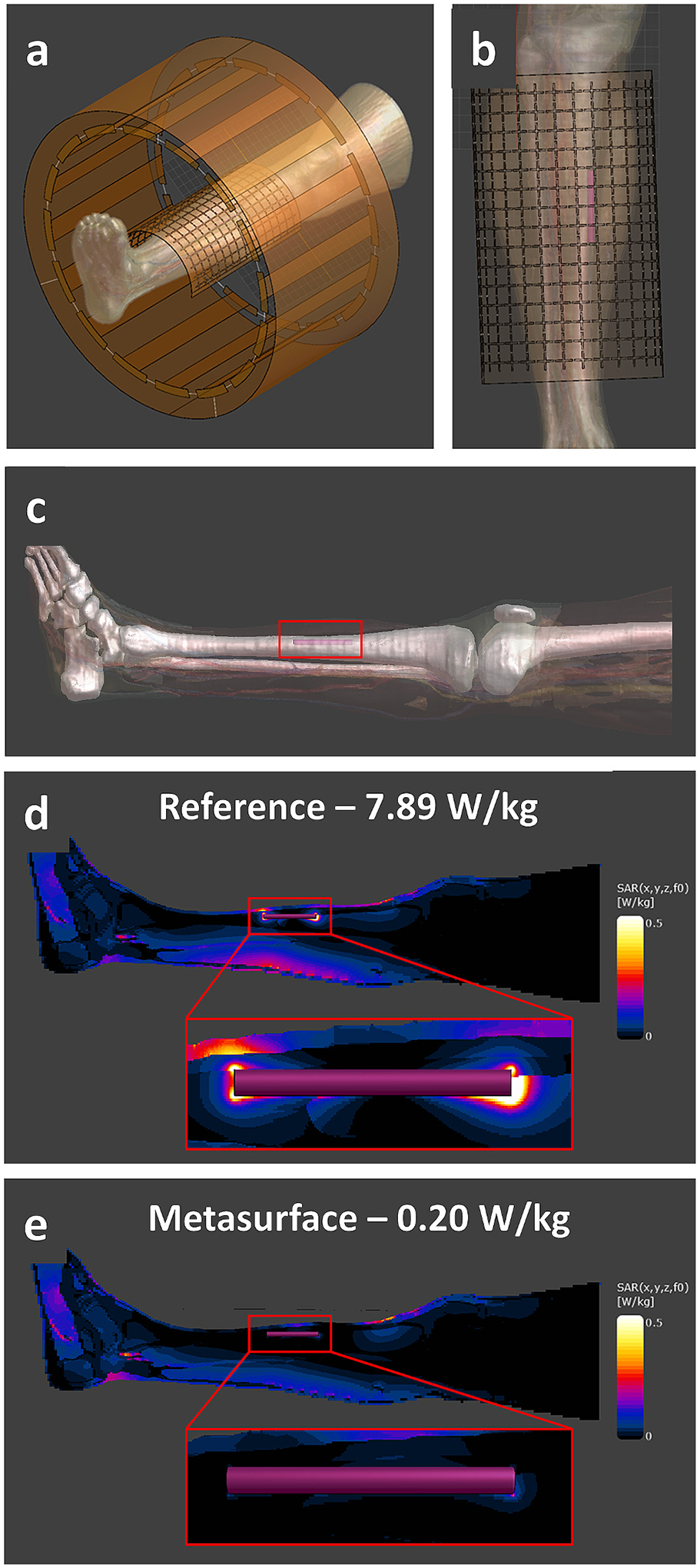
(a) The RF coil, metasurface, human leg, and stainless-steel rod models in the Sim4Life environment. (b) The positioning and alignment of the metasurface relative to the leg. The stainless-steel rod (pink cylinder) was placed in a position where it would be both near the center of the tibia and metasurface. (c) A side view showing the anterior placement of the stainless-steel rod in the tibia bone (red outline). (d) SAR_1g_ distribution maps of the entire calf under reference conditions (without the metasurface present) with an inset showing the distribution near the distal ends of the stainless-steel rod. (e) The same SAR_1g_ map and inset with the metasurface present. For both maps, psSAR values are displayed which were observed near the stainless-steel rod. It should be noted that the SAR_1g_ maps are displayed sagittally from the direct center of the simulated leg and stainless-steel rod model showing the same region with the maximum SAR_1g_ values.

The phantom model and simulation environment can be seen in Fig. 5a with a cross-section of the model shown in Fig. 5b. Simulated SAR_1g_ maps can be seen in Fig. 5c showing the effects of both the metasurface and stainless-steel rod on the SAR distribution. A clear decrease in SAR magnitude can be observed near the distal ends of the rod when the metasurface is present. The psSAR values also reflected this trend with a 40.7 % decrease when the metasurface was present (2.55 W/kg to 1.51 W/kg), correlating closely with the empirical temperature data. A comparison of results from the simulated reference, metasurface, and copper sheet can be seen showing the ${\text{B}}_{1}^{+}$ and E field distributions (Fig. 6c). Here it can be observed that the metasurface produces a small local decrease in the ${\text{B}}_{1}^{+}$ field immediately below it, in comparison to the reference condition, while the copper sheet decreases the ${\text{B}}_{1}^{+}$ field in a much larger area directly beneath it. Similar results are seen with the E field distribution, where the metasurface reduces much of the E field in the same area as with the ${\text{B}}_{1}^{+}$ field while the copper sheet produces a much larger decrease. Plots showing the field values along the 1-dimensional line profile can be seen for ${\text{B}}_{1}^{+}$ (Fig. 6d) and E fields (Fig. 6e). Here a lower minimum ${\text{B}}_{1}^{+}$ value can be seen for the metasurface at approximately 23 mm compared to the copper sheet, while E field values at the same depth are approximately equal. In comparison to the reference condition both the metasurface and copper sheet substantially decreased the E field values at 23 mm. Additional simulations were also performed using a human leg model with the same stainless steel rod partially embedded in the anterior tibia bone with the metasurface placed on top as seen in Fig. 7a, b, and c. Simulated SAR_1g_ maps of the leg and stainless steel rod show a reduction in SAR distribution in the posterior region of the calf and more substantially in the local area around the implant. Quantitatively, the psSAR value around the implant was 7.89 W/kg in the reference configuration (Fig. 7d) and decreased to 0.20 W/kg (97.4 % decrease) with the metasurface present (Fig. 7e).

### 4.4. Phantom image quality

**Fig. 8.**
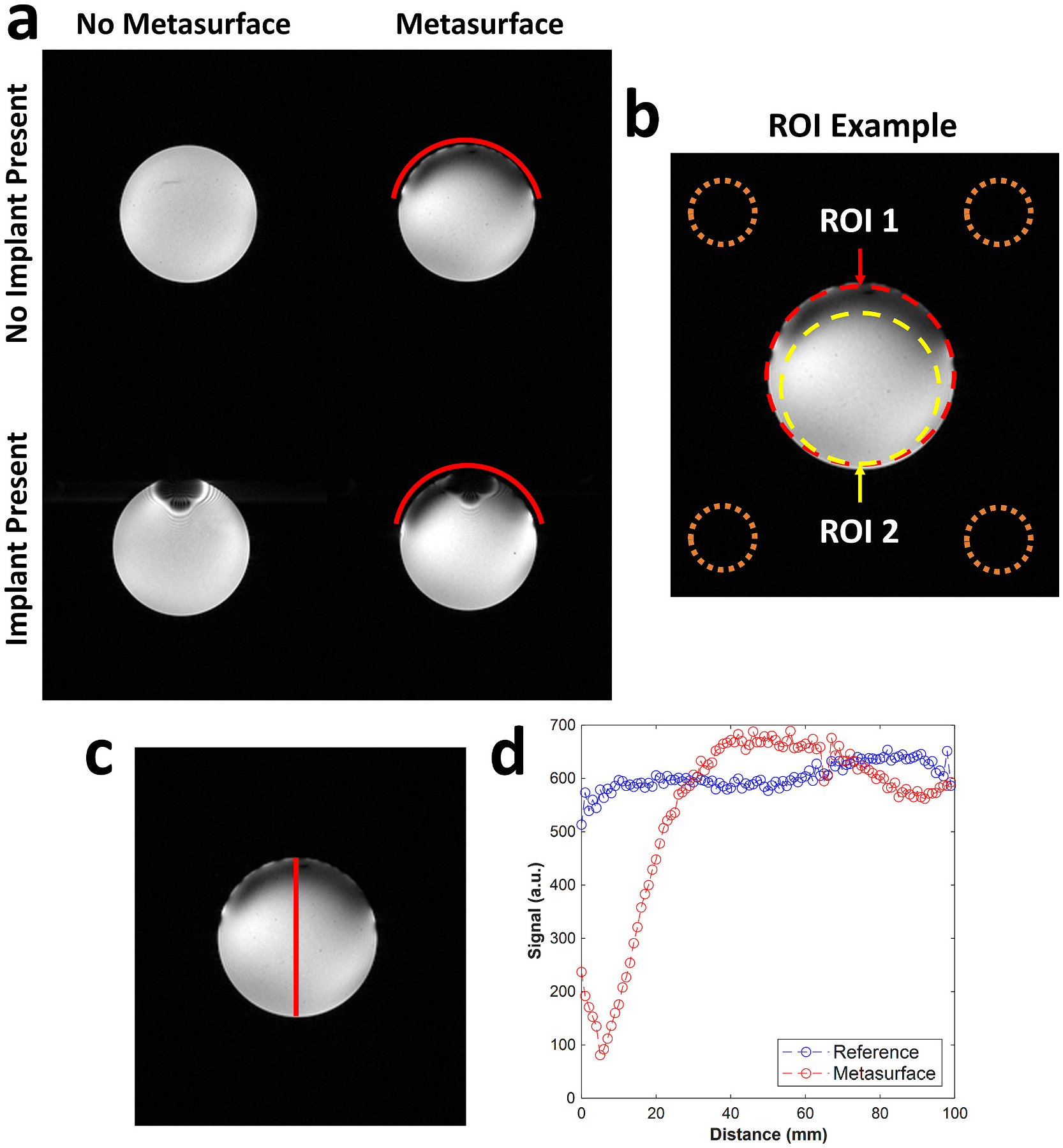
(a) Anatomic style PAA phantom images showing the effect of the metasurface (position denoted by red line) on the image quality in cases where a stainless-steel 46 mm orthopedic screw is present and absent directly under the metasurface placement. (b) SNR quantification ROIs denoted as ROI 1 (red outline) and ROI 2 (yellow outline) to delineate the effect that the metasurface has within its immediate vicinity versus unaffected regions of the images. Background noise was calculated as an average from all four of the orange outlined regions in each corner of the image. (c) The phantom image with a vertical line profile placed at the center to analyze metasurface penetration depth. (d) A plot between the phantom line profile and image intensity showing the depth at which the signal intensity was at 50 % was approximately 20 mm below the inner phantom case. In this plot the distance was defined as the distance from the metasurface.

Images acquired on the PAA phantom, seen in Fig. 8a, showed the effects that both the metasurface and a 46 mm orthopedic screw have on image quality and SNR in ROI 1 and ROI 2 (Fig. 8b). With both the metasurface and screw absent the SNR in ROI 1 was 152 and used as a reference for relative SNR calculations. When the metasurface was incorporated the SNR decreased to 139 and the relative SNR to 0.91. Incorporating the screw alone showed a similar effect by producing the lowest SNR at 116 and relative SNR of 0.76. When both the metasurface and screw were present in the phantom the SNR was 122 with a relative SNR of 0.80, similar to the SNR observed with the screw alone. In ROI 2, when the metasurface and screw were absent the SNR was 157 and used as a reference for the relative SNR under this ROI condition. When the metasurface was incorporated the SNR decreased slightly to 153 with a relative SNR of 0.97. Incorporating the screw alone had a similar effect, producing the lowest SNR of 125 and a relative SNR of 0.79. When both the metasurface and screw were present the SNR was 135 with a relative SNR of 0.86. Both SNR and relative SNR values can all be seen summarized in Table 1. To analyze the effective range of the metasurface on image quality, image intensity was plotted relative to a vertical 1-dimensional line profile (Fig. 8c). This can be seen in Fig. 7d showing the depth at which approximately 50 % of the signal decreases to be approximately 20 mm below the acrylic phantom case.

### 4.5. In-Vivo calf image quality

**Fig. 9.**
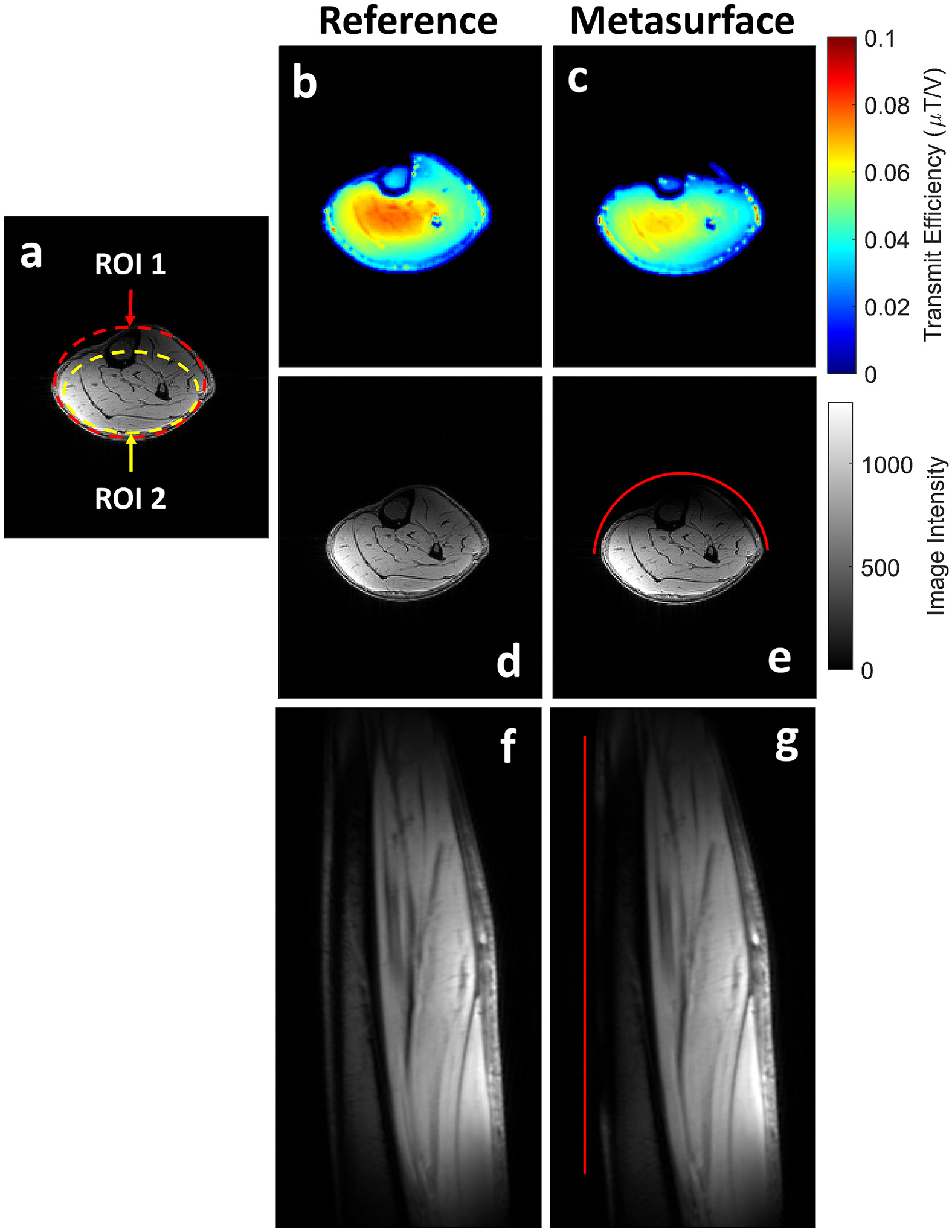
(a) ROI 1 (red outline) and ROI 2 (yellow outline) regions used to quantify SNR overall and outside the immediate vicinity of the metasurface for in-vivo axial calf images. Transmit efficiency (TE) maps can be seen with (c) and without (d) the metasurface present. Axial GRE images with (e) and without (d) the metasurface present with ROI 1 and ROI 2 SNR quantification regions shown. Corresponding sagittal GRE images can also be seen with (g) and without (f) the metasurface present. The in-vivo placement of the metasurface for both axial and sagittal images is shown by the red line placed anterior to the calf.

Images acquired on a healthy volunteer, seen in Fig. 9, show the effect of the metasurface on in-vivo image quality. Axial transmit efficiency maps can be seen for both reference and metasurface conditions, seen in Fig. 9b and c, showed that the metasurface reduced the overall transmit efficiency across the calf slice. This was also reflected in the 9.7 % decrease in average transmit efficiency across the slice (0.041 to 0.037μT/V). For the axial anatomic GRE image quality, the SNR in ROI 1 under the reference condition (Fig. 9d) was 185 and decreased to 176 (relative SNR = 0.95) with the metasurface present (Fig. 9e). Similar changes were observed in ROI 2 where, under the reference condition, the local SNR was 226 and decreased to 217 (relative SNR = 0.96). SNR values can be seen summarized in Table 1. Additionally, sagittal GRE images can also be seen for the reference (Fig. 9f) and metasurface (Fig. 9g) conditions, showing qualitatively that the metasurface (red line) placed immediately anterior to the tibia, only decreases the image quality in its immediate vicinity.

## 5. Discussion

The aim of this work was to demonstrate a metasurface tailored to locally reduce the RF field in a region containing an orthopedic metallic implant at 7 T, thereby reducing RF heating and improving safety. We also showed that image quality was not substantially affected outside the immediate vicinity of the metasurface, both in a phantom and in-vivo. The goal of this work was to make metallic implants compatible with sequences such as chemical exchange saturation transfer (CEST) based imaging, which have a SAR level similar to what was used here [31]. The empirical results presented here showed that under a 20-min, high SAR exposure, the metasurface was able to reduce the resulting maximum temperature of both implants tested by approximately 0.43 °C for the 46 mm screw (32 % decrease) and 0.57 °C for the 76 mm screw (37 % decrease), demonstrating basic effectiveness of the approach. The implants chosen for this study were positioned superficially and fixed to bone, meaning that the implants fall within the effective spatial range of the metasurface (~20 mm), and that torque induced by the Lorentz forces are less of a concern compared to other types of implants such as aneurysm clips. Additionally, since extremity imaging currently represents a large portion of imaging being performed at 7 T, orthopedic bone screws are particularly relevant for assessing effectiveness with the metasurface.

In the phantom simulations the decrease in psSAR value (~40 %) showed good agreement with the average physical temperature decreases observed when the metasurface was present versus the reference condition. These simulated results provide complementary information by illustrating the approximate change in the spatial SAR distribution rather than a single-point temperature measurement near the implant. This data would suggest that since *E*-field is proportional to SAR, that both the ${\text{B}}_{1}^{+}$ and the E-field distributions are being altered towards a lower magnitude when the metasurface is present, possibly due to lower total energy coupling with the phantom volume. Additionally, the physical transmit efficiency distribution maps also support this by showing that the metasurfaces do not produce any major unintended regions of high magnitude outside of the intended effective area that would detrimentally alter the global SAR. Simulations comparing the metasurface to a continuous flexible conductor (copper sheet) showed that at the 23 mm depth (approximately where the implant is placed) the metasurface produced a more localized area with a ${\text{B}}_{1}^{+}$ magnitude value lower than the copper sheet. *E* field values showed altered distributions between experimental conditions with the metasurface having a marginally lower value at 23 mm compared to the copper sheet, and both have a substantially lower value compared to the reference. When assessing the human leg model simulations, the metasurface substantially lowered the psSAR value by approximately 97 %. One potential reason why the human leg model showed a much higher SAR attenuation than the phantom model could be due to the metallic implant being mostly embedded in bone which has much different conductivity that the homogenous phantom PAA material as well as having a different effective wavelength, which results in substantially different SAR [6].

When assessing image quality, it was seen that while the metasurface alone reduced the relative SNR by 8.5 % compared to the reference phantom, it did not reduce it to the same degree as the implant alone (23.6 %). Furthermore, when the metasurface and implant were incorporated together in the phantom the relative SNR was only reduced by 19.7 %. A similar trend in SNR was also seen in the smaller ROI 2 that was positioned outside the metasurface range. However, when comparing ROI 1 and ROI 2 measures, the ROI 2 consistently had higher absolute and relative SNR values, supporting the notion that using this metasurface tailored to reduce RF transmission within a local area that already shows implant related image degradation, would preserve image quality outside the metasurface’s effective range. This is also supported by the use of the metasurface during in-vivo calf imaging. When the metasurface was placed anteriorly to the calf the only anatomical region which showed substantial intensity reduction was around the tibialis anterior. Both ROI 1 and ROI 2 only showed a 4.8 % drop in relative SNR, supporting practical in-vivo usage in areas outside the effective range of the metasurface.

An example of a previous study that aimed to use a similar technique to shield implantable devices, includes the work by Yang et al. [27] in which they developed an absorbing RF shield (primarily composed of a copper sheet) to reduce RF-induced heating of various DBS lead trajectories at the 1.5 T Larmor frequency. Another example was by Park et al. [26] who used a single large secondary resonator loop to produce an opposing magnetic field, like those described here, to partially shield a metal containing phantom at the 3 T Larmor frequency, thereby reducing RF-induced heating and improving implant safety. While both designs were able to effectively reduce RF-induced heating, they were only tested at lower frequencies in external RF coils without the use of image-based experiments to assess the effects on in-vivo image quality, as was done here. Additionally, while it is feasible that a continuous flexible conductor, such as the copper sheet used by Yang et al. [27] or simulated here in the present work, could function as a shield in a similar manner to the metasurface, there are a few notable challenges. First, a copper sheet cannot be tuned in the same manner as a metasurface and therefore the degree of shielding (or enhancement) cannot be modulated. Second, if a large enough copper sheet is used then eddy current formation may result in direct heating of the metal, which could then transfer that heat to the surrounding tissues. Lastly, because a bulk piece of copper metal is being used metal artifacts are expected to be worse in terms of image quality compared to the metasurface.

Considering that many medical device manufacturers have not yet tested implant compatibility with ultra-high field strength systems [32], there is currently a need for more rigorous testing as well as for an easy method towards improving safety under these conditions. Some groups have investigated safety at ultra-high fields, such as Kraff et al. [33], who showed cranial fixation plates to be relatively safe when undergoing conventional imaging sequences, with minimal impact on the permissible RF power levels. However, when considering more SAR-intensive sequences one may benefit from using the current metasurface as an additional safety measure, until sufficient simulated or empirical evidence is produced.

Since the metasurfaces were seen to be effective for small passive implants in their vicinity, it’s expected that this same effectiveness would not be fully translatable to larger implants, implying current applicability towards implants located superficially in-vivo. Another limitation included a small variety of small orthopedic implants being tested here as opposed to larger implants such as hip replacements, plates, nails, and joints which may not be compatible with the initial metasurface design presented here. Further simulations and empirical validation studies could establish improved metasurface models that would enable further tailoring of the RF field. Also, additional theoretical modeling efforts could help elucidate the RF field effects in more detail and help identify design and modeling guidelines, for example related to the desired separation thickness between the metasurface and subject [34,35]. From an image quality point of view, while the metasurfaces were able to preserve image quality in areas away from the metasurface vicinity, the tissue immediately adjacent to the metasurface saw substantial SNR degradation. To this point it is important to note that these local proximity areas would already be degraded by metal artifacts from the implant to be shielded. In this way the metasurface is primarily applicable in situations where imaging is desired in regions sufficiently far away from the metasurface. Finally, our simulations only used a generic stainless-steel rod in a PAA phantom and human leg model, whereas a more diverse palate of implant models would allow for better delineation of the nuances in the effects of the metasurface.

Future directions for this work include investigation into precise control of the field reduction effects at larger distance from the metasurface to transfer this approach to other implant types and applications, such as cardiac stents, DBS leads, and craniofacial implants. Since the in-vivo imaging presented here was performed on a subject without an implant present, additional imaging experiments on a subject with a metallic implant would allow for full evaluation of image quality changes under realistic conditions. Lastly, with pTx techniques becoming an increasingly common method for correcting ${\text{B}}_{1}^{+}$ in-homogeneities, additional work comparing the separate and combined compatibilities between this type of metasurface design and pTx RF coils is warranted.

## 6. Conclusion

In this work we introduced a metasurface design optimized to locally reduce the ${\text{B}}_{1}^{+}$ field and *E*-field as well as the associated RF-induced heating from passive metallic orthopedic implants. Empirical temperature measurements and electromagnetic simulations in a cylindrical phantom and human leg model showed a substantial decrease in local SAR around the implant. Phantom and in-vivo calf imaging also showed relatively small changes in relative SNR outside the metasurface effective range. While these current results may not directly be translatable to larger or more centrally located implants, the metasurface showed a high degree of effectiveness in reducing the amount of heating in the small implants tested, which can be generalized to other implants of similar size and physical placement.

## Figures and Tables

**Table 1:** Signal-to-noise (SNR) values for both phantom and in vivo calf imaging experiments with and without metasurfaces in ROI 1 and ROI 2 visualized in the corresponding figures. SNR values for both implant and no implant conditions can be observed for the phantom image experiment with relative SNR seen in parentheses. It should be noted that the relative SNR normalization was performed by using the no metasurface – no implant SNR value as the reference for the respective ROI regions.

	No Metasurface SNR (relative SNR)		Metasurface SNR (relative SNR)	
	ROI 1	ROI 2	ROI 1	ROI 2
Phantom – No implant	152 (1.00)	157 (1.00)	139 (0.91)	153 (0.97)
Phantom – Implant	116 (0.76)	125 (0.79)	122 (0.80)	135 (0.85)
In vivo calf	185	226	176	217

## Data Availability

Data outlined in this study are available from the corresponding author, upon reasonable request.
